# Human midbrain organoids reveal the characteristics of axonal mitochondria specific to dopaminergic neurons

**DOI:** 10.1186/s13041-025-01268-w

**Published:** 2025-12-25

**Authors:** Akihiko Nishijima, Mutsumi Yokota, Soichiro Kakuta, Akihiro Yamaguchi, Kei-ichi Ishikawa, Hideyuki Okano, Wado Akamatsu, Nobutaka Hattori, Masato Koike

**Affiliations:** 1 Department of Cell Biology and Neuroscience, Juntendo University Graduate School of Medicine, Tokyo, 113-8421 Japan; 2 Laboratory of Morphology and Image Analysis, Biomedical Research Core Facilities, Juntendo University Graduate School of Medicine, Tokyo, 113-8421 Japan; 3 Center for Genomic and Regenerative Medicine, Juntendo University Graduate School of Medicine, Tokyo, 113-8421 Japan; 4 Department of Neurology, Faculty of Medicine, Juntendo University, Tokyo, 113-8421 Japan; 5 Keio University Regenerative Medicine Research Center, Kanagawa, 210-0821 Japan; 6 Neurodegenerative Disorders Collaboration Laboratory, RIKEN Center for Brain Science, Saitama, 351-0198 Japan

**Keywords:** Axonal mitochondria, Dopaminergic neurons, Midbrain organoids, Live-cell imaging, Electron microscopy

## Abstract

Mitochondrial dysfunction and abnormalities in mitochondrial quality control contribute to the development of neurodegenerative diseases. Parkinson’s disease is a neurodegenerative disease that causes motor problems mainly due to the loss of dopaminergic neurons in the substantia nigra pars compacta. Axonal mitochondria in neurons reportedly differ in properties and morphologies from mitochondria in somata or dendrites. However, the function and morphology of axonal mitochondria in human dopaminergic neurons remain poorly understood. To define the function and morphology of axonal mitochondria in human dopaminergic neurons, we newly generated tyrosine hydroxylase (*TH*) reporter (*TH*-GFP) induced pluripotent stem cell (iPSC) lines from one control and one *PRKN*-mutant patient iPSC lines and differentiated these iPSC lines into dopaminergic neurons in two-dimensional monolayer cultures or three-dimensional midbrain organoids. Immunostainings with antibodies against axonal and dendritic markers showed that axons could be better distinguished from dendrites of dopaminergic neurons in the peripheral area of three-dimensional midbrain organoids than in two-dimensional monolayers. Live-cell imaging and correlative light–electron microscopy in peripheral areas of midbrain organoids derived from control *TH*-GFP iPSCs demonstrated that axonal mitochondria in dopaminergic neurons had lower membrane potential and were shorter in length than those in non-dopaminergic neurons. Although the mitochondrial membrane potential did not significantly differ between dopaminergic and non-dopaminergic neurons derived from *PRKN*-mutant patient lines, these differences tended to be similar to those in control lines. These results were also largely consistent with those of our previous study on somatic mitochondria. The findings of the present study indicate that midbrain organoids are an effective tool to distinguish axonal from dendritic mitochondria in dopaminergic neurons. This may facilitate the analysis of axonal mitochondria to provide further insights into the mechanisms of dopaminergic neuron degeneration in patients with Parkinson’s disease.

**Supplementary Information:**

The online version contains supplementary material available at 10.1186/s13041-025-01268-w.

## Introduction

Mitochondrial function and morphology are associated with the regulation of cellular energy metabolism, redox signaling, apoptosis, and cell fate [1, 2]. Abnormalities in mitochondrial function and morphology, as well as failure of mitochondrial quality control, contribute to neurodegenerative diseases [3]. Parkinson’s disease (PD) is a neurodegenerative disease characterized by symptoms of bradykinesia, tremor, and muscular rigidity [4]. The gene *PRKN* (also known as *PARK2*), which is associated with early-onset familial PD, encodes the parkin RBR E3 ubiquitin protein ligase [5], which plays an important role in mitochondrial quality control [5–7]. PD patients with *PRKN* mutations exhibit preferential loss of dopaminergic neurons in the substantia nigra pars compacta [4]. However, the detailed mechanisms resulting in the preferential degeneration of dopaminergic neurons in patients with PD remain unclear.

To analyze mitochondrial function and morphology specifically in dopaminergic neurons of patients with PD, we have previously established tyrosine hydroxylase (*TH*; a marker for identifying dopaminergic neurons) green fluorescence protein (GFP) reporter lines from control induced pluripotent stem cell (iPSC) lines and a patient iPSC line with a *PRKN* mutation via the CRISPR/Cas9 system [8]. Correlative light–electron microscopy (CLEM) analysis and live-cell imaging using these *TH*-GFP iPSC lines demonstrated that somata of GFP-positive dopaminergic neurons had smaller mitochondria with lower membrane potential than somata of GFP-negative non-dopaminergic neurons [8].

In dopaminergic neurons, the morphology and function of mitochondria in axons probably differ from those in somata and dendrites. Several studies using rodent hippocampal and cortical neurons have reported that mitochondria in somata and dendrites are tubular, whereas those in axons are small and discrete [9–11]. In rodent hippocampal neurons, most of the generated ATP is required for the synaptic vesicle cycle at the presynaptic terminal [12], and mitochondria-regulated higher presynaptic Ca^2+^ levels are needed for synaptic vesicle release [13, 14]. To understand mitochondrial abnormalities in the entirety of dopaminergic neurons from patients with PD, it is crucial to analyze axonal mitochondria in dopaminergic neurons. However, only a few studies have examined axonal mitochondria in human dopaminergic neurons.

In this study, we generated new *TH*-GFP iPSC lines to increase the number of such iPSC lines. Human midbrain organoids embedded in a three-dimensional (3D) matrix are a strong tool for differentiation and maturation into midbrain tissue [15–17]. Therefore, we used *TH*-GFP iPSC-derived midbrain organoids as an in vitro 3D model of mature dopaminergic neurites to investigate the function and morphology of axonal mitochondria. Most of the neurites in the peripheral area of the organoids were axons. CLEM analysis and live-cell imaging in the peripheral area of these organoids showed that axons of GFP-positive dopaminergic neurons had shorter mitochondria with lower membrane potential compared to GFP-negative non-dopaminergic neurons. This study provides evidence that *TH*-GFP iPSC-derived midbrain organoids are effective tools for studying axonal mitochondria specific to dopaminergic neurons.

## Methods

### Human iPSCs

JB6, a control iPSC line, and PH13, an iPSC line derived from a patient with PD carrying a *PRKN* mutation (exon 3, homozygous deletion), were previously reported [18, 19]. The *TH*-GFP iPSC lines 201B7 T1-3, WD39 T1-2, and PB2 T1-1 were previously established from original iPSC lines as follows [8]. The control iPSC line 201B7 was kindly provided by Dr. Shinya Yamanaka at Kyoto University [20]. WD39, another control line, and PB2, a PD patient line with a *PRKN* mutation (exon 6, 7 homozygous deletions), were previously reported [21].

### Generation of *TH*-GFP iPSC lines

The iPSC lines JB6 and PH13 were cultured on plates coated with iMatrix-511 (Nippi Inc., Tokyo, Japan) and expanded in StemFit AK02N medium (Ajinomoto, Tokyo, Japan). On day 7 after reseeding, 1 × 10^6^ cells were electroporated at 125 V for 5 ms using a NEPA21 electroporator (NEPAGENE Co., Ltd., Chiba, Japan) with 5 μg of Cas9 vectors (Addgene plasmid #60,599; a kind gift from Dr. Akitsu Hotta) [22], single-guide RNA (sgRNA) vectors [8], and donor vectors [8]. On day 2 after electroporation, the iPSCs were treated with 0.75 μg/mL of puromycin for 14 days. Puromycin-resistant colonies were manually picked for PCR screening and expansion. The colonies for expansion were dissociated using TrypLE Select (Thermo Fisher Scientific, Waltham, MA, USA) and were then cultured with CloneR (StemCell Technologies Inc., Vancouver, BC, Canada).

### PCR screening

Knock-in clones were identified according to the previously reported screening method for *TH*-GFP iPSC clones [8]. PCR primers for the detection of the *TH*-GFP allele (forward primer, 5′-ACT GCC TGT CTG AGG AGC CTG AGA TTC-3′; reverse primer, 5′-TTG CTG CGG ATG ATC TTG TCG GTG AAG ATC-3′) were designed to cover the *TH* gene and the GFP sequence.

### Differentiation of *TH*-GFP iPSCs into dopaminergic neurons in two-dimensional (2D) monolayer culture

*TH*-GFP iPSCs were cultured on plates coated with iMatrix-511 (Nippi Inc.) and expanded in StemFit AK02N medium (Ajinomoto). Subsequently, *TH*-GFP iPSCs were differentiated into dopaminergic neurons using the previously established direct neurosphere converting method [23–26]. Briefly, *TH*-GFP iPSCs were cultured using the CTraS method (cultured in AK02N medium containing 3 μM CHIR99021, 3 μM dorsomorphin, and 3 μM SB431542 [all Nakalai Tesque, Kyoto, Japan]) for 6 days. The dissociated iPSCs were then cultured in suspensions in KBM Neural Stem Cell medium (Kohjin Bio Co., Ltd., Saitama, Japan) supplemented with B27 (Thermo Fisher Scientific), 20 ng/mL basic fibroblast growth factor (PeproTech, Cranbury, NJ, USA), 2 μM SB431542 (Nakalai Tesque), and 10 μM Y27632 (Fujifilm Wako Pure Chemical Corporation, Osaka, Japan) at 4% O_2_ to form neurospheres. After 3 days of suspension culture, CHIR99021 (final concentration 3 μM) and purmorphamine (final concentration 2 μM, Millipore Corporation, Burlington, MA, USA) were added to the medium. After 13 days of suspension culture, neurospheres were dissociated and reseeded onto coverslips coated with poly-L-ornithine (Sigma-Aldrich, St. Louis, MO, USA) and fibronectin (Corning Inc., Corning, NY, USA). For terminal differentiation, the dissociated cells were cultured for 9 days in KBM Neural Stem Cell medium containing B27, 20 ng/mL brain-derived neurotrophic factor (BioLegend, San Diego, CA, USA), 20 ng/mL glial cell-derived neurotrophic factor (Alomone Labs, Jerusalem, Israel), 0.2 mM ascorbic acid (Sigma-Aldrich), 0.5 mM dibutyryl-cAMP (Nakalai Tesque), 1 ng/mL transforming growth factor beta 3 (BioLegend), and 10 μM DAPT (Sigma-Aldrich). For the first 3 days of terminal differentiation, the cells were cultured in terminal differentiation medium further supplemented with CHIR99021 at a final concentration of 3 μM.

### Differentiation of *TH*-GFP iPSCs into 3D midbrain organoids

*TH*-GFP iPSC-derived midbrain organoids were generated as reported previously [15–17]. *TH*-GFP iPSC-derived neurospheres cultured in suspension for 8 days were dissociated and seeded at 1 × 10^4^ cells per well into ultralow-attachment round-bottom 96-well plates (Fujifilm). The cells were cultured in KBM Neural Stem Cell medium supplemented with B27, 20 ng/mL basic fibroblast growth factor, 2 μM SB431542, 10 μM Y27632, 3 μM CHIR99021, 2 μM purmorphamine, and 0.2 mM ascorbic acid for 7 days at 4% O_2_. The neurospheres were embedded in 10–15 µL droplets of Geltrex (Gibco, Grand Island, NY, USA) and cultured for 14 days in ultralow-attachment 6-well plates (Corning Inc.) in KBM Neural Stem Cell medium supplemented with B27, 20 ng/mL brain-derived neurotrophic factor, 20 ng/mL glial cell-derived neurotrophic factor, 0.2 mM ascorbic acid, 0.5 mM dibutyryl-cAMP, 1 ng/mL transforming growth factor beta 3, 10 μM DAPT, and 2.5 ng/mL activin A (PeproTech). The organoids were then matured for an additional 14 days in Neurobasal Plus Medium (Gibco) supplemented with B27 Plus (Gibco), CultureOne (Gibco), 20 ng/mL brain-derived neurotrophic factor, 20 ng/mL glial cell-derived neurotrophic factor, 0.2 mM ascorbic acid, 0.5 mM dibutyryl-cAMP, 1 ng/mL transforming growth factor beta 3, 10 μM DAPT, and 2.5 ng/mL activin A.

### Immunofluorescence staining of monolayer dopaminergic neurons and midbrain organoids

For the immunostaining of monolayer dopaminergic neurons, differentiated cells 9 days after reseeding were fixed with 4% paraformaldehyde in phosphate-buffered saline (PBS) for 10 min and permeabilized with 0.1% Triton X-100 in PBS for 15 min at room temperature. The cells were then blocked with 2% bovine serum albumin (BSA) in PBS for 30 min, stained with primary antibodies for 2 h at room temperature, washed, and finally stained with secondary antibodies and 4′,6-diamidino-2-phenylindole (DAPI; Thermo Fisher Scientific; 1:10,000) for 1 h at room temperature.

Midbrain organoids were immunostained as reported previously [16]. The midbrain organoids were fixed in 4% paraformaldehyde in 0.1 M phosphate buffer for 3 h at room temperature, washed three times with PBS, and embedded in 3% agarose. The agarose blocks were cut into 100-µm sections using a Linear Slicer PRO7N (Dosaka EM Co., Ltd., Kyoto, Japan). The sections were blocked with 0.5% Triton X-100, 2% BSA, and 5% normal donkey serum in PBS for 90 min at room temperature. Subsequently, the sections were stained with primary antibodies diluted in PBS containing 0.1% Triton X-100, 2% BSA, and 5% normal donkey serum for 72 h at 4 °C. After washing three times with PBS, the sections were stained with secondary antibodies and DAPI diluted in PBS containing 0.1% Triton X-100, 2% BSA, and 5% normal donkey serum for 2 h at room temperature, followed by three washes with PBS and one wash with distilled water. The sections were mounted in Fluoromount-G Mounting Medium (Southern Biotech, Birmingham, AL, USA).

The following primary antibodies were used: mouse anti-GFP antibody (MBL Co., Ltd., Nagoya, Japan; 1:500), rabbit anti-TH antibody (PelFreez Biologicals, Rogers, AR, USA; 1:400), mouse anti-Tau antibody (Abcam, Cambridge, MA, USA; 1:1000), and goat anti-microtubule-associated protein (MAP)2 antibody (Abcam; 1:200). The following secondary antibodies were used: Alexa Fluor 488-, 555-, and 647-conjugated secondary antibodies (Thermo Fisher Scientific; 1:800). Immunostaining images were taken using a confocal microscope (Zeiss LSM880; Carl Zeiss AG, Oberkochen, Germany).

### Live-cell imaging of monolayer dopaminergic neurons and midbrain organoids

To measure mitochondrial membrane potential, monolayer dopaminergic neurons were reseeded on μ-dishes (ibidi GmbH, Gräfelfing, Germany) and incubated with 20 nM tetramethylrhodamine methyl ester (TMRM) and 100 nM MitoTracker Deep Red (both Thermo Fisher Scientific) for 30 min at 37 °C. The images were taken using a confocal microscope (Zeiss LSM880). The mean fluorescence intensity (MFI) of TMRM in monolayer dopaminergic neurons was measured by encircling MitoTracker Deep Red-positive (intensity ≥ 30) mitochondria with ZEN software.

Midbrain organoids were attached to Geltrex-coated μ-dishes (ibidi GmbH) overnight and incubated with 20 nM TMRM for 30 min at 37 °C. Because we were unable to stain organoids with MitoTracker Deep Red, possibly due to its poor penetration into the organoids, we used TMRM fluorescence itself as an indicator of mitochondria. The z-stack images were taken using a confocal microscope (Zeiss LSM880) and processed using a maximum-intensity projection. Because we were unable to use the mitochondrial indicator MitoTracker, the MFI of TMRM in midbrain organoids was measured by encircling TMRM-positive mitochondria (intensity ≥ 30, pixel size ≥ 15) with ZEN software.

### CLEM of *TH*-GFP iPSC-derived midbrain organoids

Midbrain organoids were attached to Geltrex-coated gridded coverslips (Matsunami Glass Ind., Ltd., Osaka, Japan) and stained with 20 nM TMRM for 30 min to indicate mitochondria on fluorescent images. After taking bright-field and fluorescent images using a BZ-X710 fluorescent microscope (Keyence Corporation, Osaka, Japan), the organoids were fixed in 0.1 M phosphate buffer containing 2% glutaraldehyde and 50 mM sucrose and post-fixed in 1% osmium tetroxide. Fixed organoids were dehydrated and embedded in Epok812 (Oken Shoji Co., Ltd., Tokyo, Japan). Ultrathin sections were cut using a UC6 ultramicrotome (Leica Microsystems, Wetzlar, Germany), stained with uranyl acetate and lead citrate, and observed using a JEM-1400 electron microscope (JEOL Ltd., Tokyo, Japan). In electron microscopic images of axons taken in the peripheral area of the organoids, the length of the mitochondrial major axis was blindly measured using ImageJ, and then fluorescent images were used to confirm whether the axons were GFP-positive or GFP-negative. Ten GFP-negative and six GFP-positive axons from control JB6 T1-6 organoid and five GFP-negative and four GFP-positive axons from *PRKN*-mutant PH13 T1-6 organoid were measured.

### Statistical analysis

Mitochondrial length and membrane potential were statistically analyzed using the mean value of mitochondrial length or membrane potential per neurite or axon. GraphPad Prism 10 software and Python version 3.14.0 were used for statistical analyses. Differences between groups were evaluated using the nonparametric Mann–Whitney U test or Kruskal–Wallis test with Dunn’s multiple comparison test, as appropriate. A *P*-value of less than 0.05 was considered significant.

## Results

### Establishment of new control and *PRKN*-mutant patient *TH*-GFP iPSC lines

Using the CRISPR/Cas9 system, we have previously established *TH*-GFP iPSC lines from two control iPSC lines and one *PRKN*-mutant patient iPSC line [8]. To further generalize our findings, we newly generated control and *PRKN*-mutant patient *TH*-GFP iPSC lines by electroporating the control iPSC line JB6 [18] and the *PRKN*-mutant patient iPSC line PH13 [19] with Cas9, sgRNA, and donor vectors for insertion of the GFP sequence into the Target 1 site of the *TH* gene according to our previously reported method [8]. After 14 days of puromycin selection, we picked puromycin-resistant colonies and screened for iPSC clones that carried the GFP sequence at the Target 1 site of the *TH* gene. PCR analysis detected bands representing the *TH*-GFP allele in four of eight puromycin-resistant control JB6 iPSC clones and two of eight puromycin-resistant *PRKN*-mutant PH13 iPSC clones (Fig. S1 in Additional file 1). The control JB6 T1-6 and *PRKN*-mutant PH13 T1-6 *TH*-GFP iPSC lines, which showed high differentiation into dopaminergic neurons, were then used for further experiments.

**Fig. 1:**
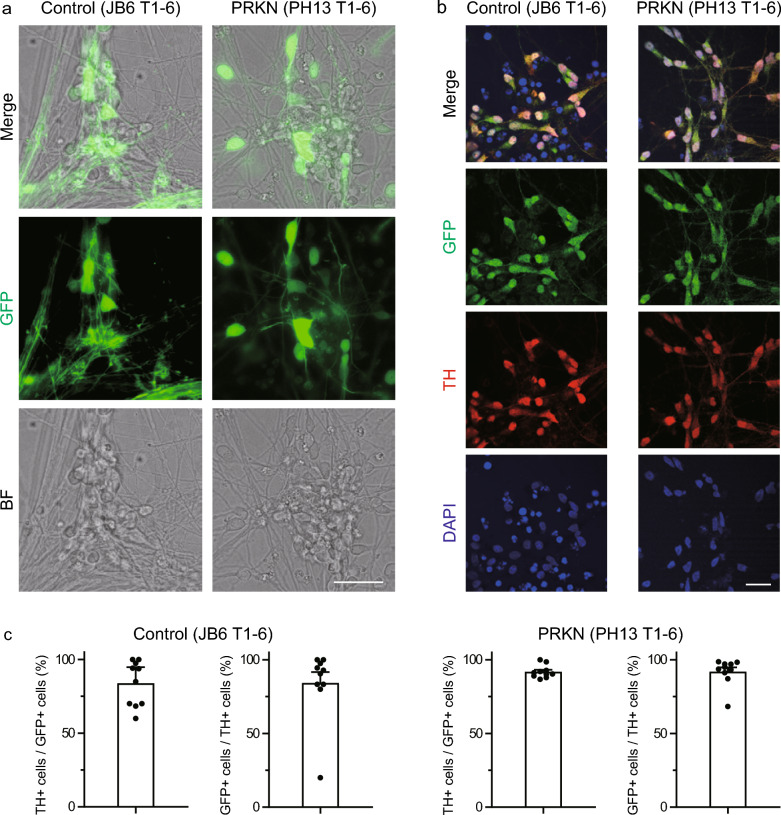
Characterization of GFP-positive neurons differentiated from the generated control JB6 T1-6 and *PRKN*-mutant PH13 T1-6 *TH*-GFP iPSC lines. **a** GFP and BF images of *TH*-GFP iPSC-derived neurons on day 9 of differentiation after reseeding dissociated neurospheres. “PRKN” represents *PRKN*-mutant patient. Scale bar, 50 µm. **b** Immunofluorescence staining of *TH*-GFP iPSC-derived neurons with antibodies against GFP and TH on day 9 of differentiation after reseeding dissociated neurospheres. “PRKN” represents *PRKN*-mutant patient. Scale bar, 20 µm. **c** Percentage of TH-positive cells among GFP-positive cells (left) and of GFP-positive cells among TH-positive cells (right) in immunofluorescence images (n = 10 fields per iPSC line). “PRKN” represents *PRKN*-mutant patient. Values are shown as the mean ± SEM. BF, bright field; DAPI, 4′,6-diamidino-2-phenylindole; GFP, green fluorescence protein; TH, tyrosine hydroxylase

To confirm the expression of the *TH*-GFP gene in the newly established control JB6 T1-6 and *PRKN*-mutant PH13 T1-6 iPSC lines, these iPSC lines were differentiated into monolayer dopaminergic neurons using the direct neurosphere converting method as previously reported [23–26]. We confirmed that some of the differentiated neurons expressed GFP in both control JB6 T1-6 and *PRKN*-mutant PH13 T1-6 *TH*-GFP iPSC lines (Fig. 1a). Immunostaining of *TH*-GFP iPSC-derived neurons with anti-GFP and anti-TH antibodies demonstrated that GFP and TH were colocalized in control JB6 T1-6 and *PRKN*-mutant PH13 T1-6 *TH*-GFP iPSC-derived neurons (Fig. 1b). Based on the proportions of TH-positive cells among GFP-positive cells (control JB6 T1-6, 83.9%; *PRKN*-mutant PH13 T1-6, 91.9%; Fig. 1c) and GFP-positive cells among TH-positive cells (control JB6 T1-6, 84.2%; *PRKN*-mutant PH13 T1-6, 92.0%; Fig. 1c) in immunofluorescence images, the newly established *TH*-GFP iPSC lines exhibited high specificity as TH reporters, similar to that of other *TH*-GFP iPSC lines in our previous study [8]. These results suggest that the newly established control and *PRKN*-mutant patient *TH*-GFP iPSC lines can be used for dopaminergic neuron-specific analyses, in addition to our previously established *TH*-GFP iPSC lines.

### Neuronal polarity of *TH*-GFP iPSC-derived neurons in 2D monolayer cultures

**Fig. 2:**
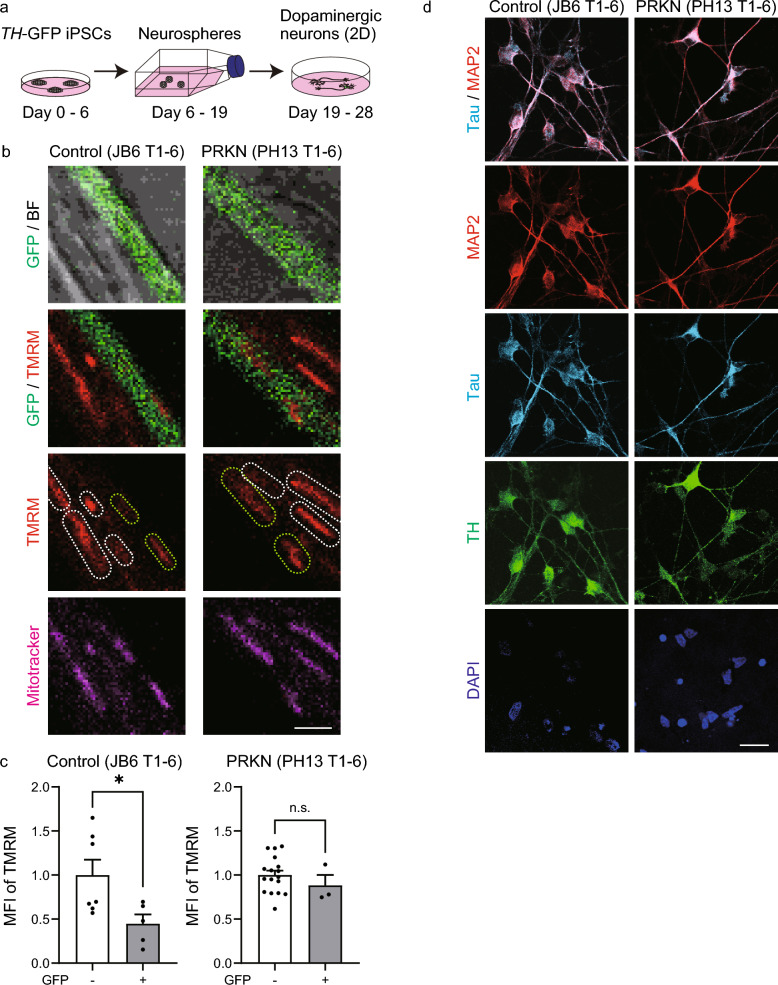
Evaluation of the neuronal polarity of *TH*-GFP iPSC-derived neurons in 2D monolayer culture for 28 days. **a** Schematic timeline of the differentiation from *TH*-GFP iPSCs into dopaminergic neurons in 2D monolayer culture. *TH*-GFP iPSCs were cultured for 6 days using the CTraS method. The *TH*-GFP iPSC-derived neurospheres were cultured in flasks for 13 days and then reseeded and differentiated into dopaminergic neurons for 9 days. **b** Live-cell imaging of neurites of dopaminergic and non-dopaminergic neurons derived from *TH*-GFP iPSC lines. Green and white dotted lines indicate mitochondria in GFP-positive and GFP-negative neurites, respectively. “PRKN” represents *PRKN*-mutant patient. Scale bar, 2 µm. **c** Mean TMRM MFI in all mitochondria per neurite from the live-cell images. Data were acquired from GFP-negative (n = 7) and GFP-positive (n = 5) neurites from three fields in the control JB6 T1-6 line and from GFP-negative (n = 17) and GFP-positive (n = 3) neurites from two fields in the *PRKN*-mutant PH13 T1-6 line. The graph shows the relative comparison of the mean TMRM MFI of mitochondria in GFP-negative neurites and GFP-positive neurites. “PRKN” represents *PRKN*-mutant patient. Values are shown as the mean ± SEM. The differences were evaluated using the nonparametric Mann–Whitney U test. *P < 0.05. The mean TMRM MFI of mitochondria significantly differs between dopaminergic and non-dopaminergic neurons in the control JB6 T1-6 line (*P* = 0.0480; effect size, 0.467; 95% CI 0.0267 to 1.09), whereas the *PRKN*-mutant PH13 T1-6 line shows no significant difference (*P* = 0.301; effect size, 0.171, 95% CI, − 0.142 to 0.306). **d** Immunofluorescence stainings of *TH*-GFP iPSC-derived neurons with antibodies against MAP2, Tau, and TH indicate that dendritic and axonal markers are colocalized in neurites of dopaminergic neurons in 2D monolayers cultured for 28 days. “PRKN” represents *PRKN*-mutant patient. Scale bar, 20 µm. 2D, two-dimensional; BF, bright field; CI, confidence interval; DAPI, 4′,6-diamidino-2-phenylindole; GFP, green fluorescence protein; iPSC, induced pluripotent stem cell; MFI, mean fluorescence intensity; MAP2, microtubule-associated protein 2; n.s., not significant; SEM, standard error of the mean; TH, tyrosine hydroxylase; TMRM, tetramethylrhodamine methyl ester

To compare mitochondrial membrane potentials between neurites of dopaminergic neurons and those of non-dopaminergic neurons, we differentiated the *TH*-GFP iPSCs into monolayer dopaminergic neurons for 28 days (Fig. 2a). Live-cell imaging was performed in *TH*-GFP iPSC-derived neurons stained with TMRM, a fluorescent probe for monitoring mitochondrial membrane potential, and MitoTracker Deep Red, an indicator of mitochondria. The results on day 28 showed that in all five *TH*-GFP iPSC lines, the TMRM fluorescence intensity in GFP-positive neurites tended to be reduced compared to that in GFP-negative neurites (Fig. 2b and Fig. S2a in Additional file 1). Quantification of the TMRM MFI confirmed that mitochondria in neurites of GFP-positive dopaminergic neurons had a significantly lower membrane potential than those in neurites of GFP-negative non-dopaminergic neurons in the newly generated control *TH*-GFP iPSC lines (Fig. 2c; control JB6 T1-6, *P* = 0.048) and in the control *TH*-GFP iPSC lines established in our previous study (Fig. S2b in Additional file 1; control 201B7 T1-3, *P* = 0.0013; control WD39 T1-2, *P* = 0.0002). The mitochondrial membrane potential did not significantly differ between GFP-positive and GFP-negative neurites in the *PRKN*-mutant patient lines (Fig. 2c; *PRKN*-mutant PH13 T1-6, *P* = 0.301; Fig. S2b in Additional file 1; *PRKN*-mutant PB2 T1-1, *P* = 0.569).

However, immunostaining of monolayer dopaminergic neurons with antibodies against the dendritic marker MAP2 and the axonal marker Tau showed colocalization of MAP2 and Tau in neurites derived from control JB6 T1-6 and *PRKN*-mutant PH13 T1-6 *TH*-GFP iPSC lines (Fig. 2d). These results indicated that it was difficult to distinguish axons from dendrites in monolayer dopaminergic neurons, at least on day 28 in neurons derived from *TH*-GFP iPSCs. A previous study has reported that neuronal polarity was established after more than two weeks of terminal differentiation from neural progenitor cells [27]. Furthermore, even if neuronal polarity is established through long-term culture, axons cannot be easily identified because neurites grow in random directions. This indicates that 2D monolayer cultures are not suitable for analyses of axons without immunocytochemistry, such as live-cell imaging or electron microscopy. Therefore, we changed the strategy to differentiate iPSCs into midbrain organoids that extend neurites radially toward the peripheral area during long-term culture and further maturation, thereby allowing the analysis of mitochondria in mature neurites of the organoids.

### Evaluation of neuronal polarity in *TH*-GFP iPSC-derived midbrain organoids

**Fig. 3:**
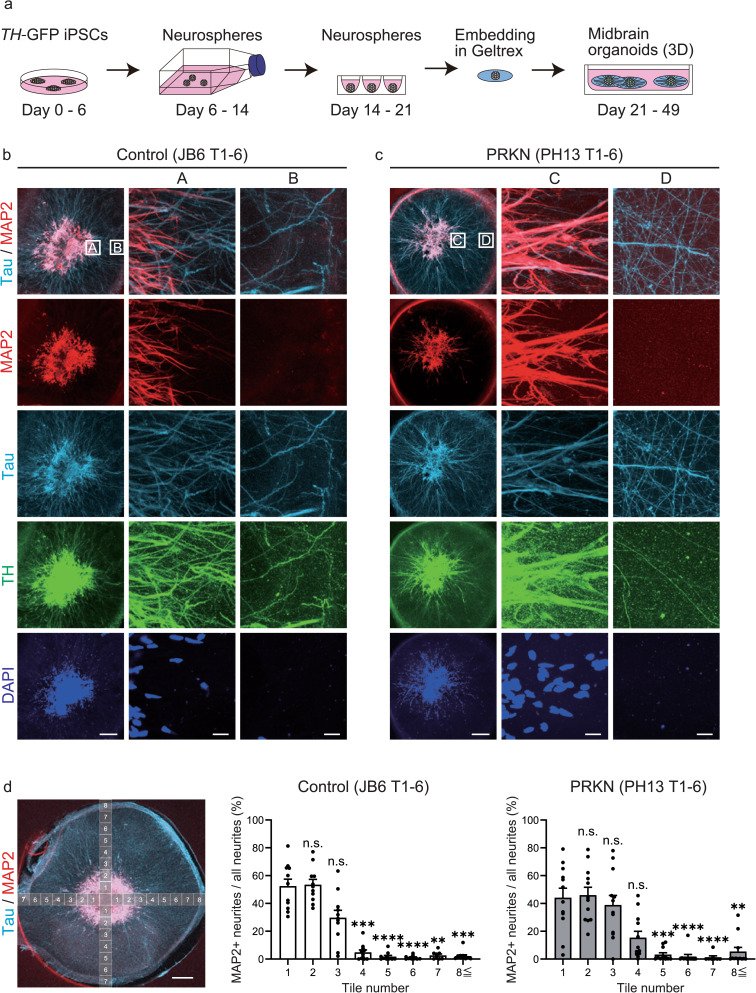
Evaluation of neuronal polarity in midbrain organoids differentiated from *TH*-GFP iPSCs for 49 days. **a** Schematic timeline of the differentiation from *TH*-GFP iPSCs into midbrain organoids. *TH*-GFP iPSC-derived neurospheres were cultured in flasks for 8 days, in ultralow-attachment plates for 7 days, embedded into Geltrex droplets, and then cultured in ultralow-attachment plates for 4 weeks. **b** Immunofluorescence stainings of control JB6 T1-6 *TH*-GFP iPSC-derived midbrain organoids with antibodies against MAP2, Tau, and TH. Scale bar of the left column, 250 µm. “A” and “B” indicate the enlarged central and peripheral areas of the left images, respectively. Scale bars of the enlarged images, 20 µm. **c** Immunofluorescence stainings of *PRKN*-mutant PH13 T1-6 *TH*-GFP iPSC-derived midbrain organoids with antibodies against MAP2, Tau, and TH. “PRKN” represents *PRKN*-mutant patient. Scale bar of the left column, 250 µm. “C” and “D” indicate the enlarged central and peripheral areas of the left images, respectively. Scale bars of the enlarged images, 20 µm. **d** Percentage of MAP2-positive neurites relative to all neurites. Fluorescent images were taken in four directions, every 134.95 µm from the core of the stained organoids for quantification, as shown in the left image. Scale bar of the left image, 500 µm. Values were calculated as the percentage of MAP2-positivite area relative to Tau and/or MAP2-positive area in fluorescence images (tile_1, n = 11; tile_2, n = 11; tile_3, n = 11; tile_4, n = 11; tile_5, n = 10; tile_6, n = 10; tile_7, n = 8; tile_8-12, n = 12 from three control JB6 T1-6 organoids, and tile_1, n = 12; tile_2, n = 12; tile_3, n = 12; tile_4, n = 12; tile_5, n = 12; tile_6, n = 11; tile_7, n = 8; tile_8-12, n = 12 from three *PRKN*-mutant PH13 T1-6 organoids). The MAP2-positive and Tau/MAP2-merged areas in the fluorescence images were measured using the thresholding algorithm of ImageJ (MAP2 threshold, 50–255; Tau/MAP2 threshold, 150–255). “PRKN” represents *PRKN*-mutant patient. Values are shown as the mean ± SEM. The differences between values in tile_1 and other tiles were evaluated using the Kruskal–Wallis test with Dunn’s multiple comparison test. **P < 0.01, ***P < 0.001, ****P < 0.0001. The percentage of MAP2-positive neurites is not significantly different between tile_1 and 2 (*P* > 0.9999) and between tile_1 and 3 (*P* > 0.9999) in the control JB6 T1-6 line, and the percentage of MAP2-positive neurites does not significantly differ between tile_1 and 2 (*P* > 0.9999), between tile_1 and 3 (*P* > 0.9999), and between tile_1 and 4 (*P* = 0.2305) in the *PRKN*-mutant PH13 T1-6 line. 3D, three-dimensional; DAPI, 4′,6-diamidino-2-phenylindole; GFP, green fluorescence protein; iPSC, induced pluripotent stem cell; MAP2, microtubule-associated protein 2; n.s., not significant; TH, tyrosine hydroxylase

We generated midbrain organoids by embedding neurospheres in Geltrex droplets and culturing them in ultralow-attachment plates for four weeks (Fig. 3a), using the methods reported in previous studies [15–17]. To assess neuronal polarity in the generated midbrain organoids, we immunostained the organoids on day 49 with antibodies against MAP2 and Tau. Immunostainings demonstrated that the central area of these organoids was composed of both MAP2- and Tau-positive neurites, whereas the peripheral area of the organoids contained almost no MAP2-positive neurites (i.e., Tau-positive neurites were predominant) in both control JB6 T1-6 (Fig. 3b) and *PRKN*-mutant PH13 T1-6 (Fig. 3c) *TH*-GFP iPSC lines. The immunostained organoids were imaged every 134.95 µm from the core to the periphery in all four directions (up, down, left, and right). Quantitative analyses of immunofluorescence images revealed that the ratio of MAP2-positive neurites to all neurites in the peripheral area of the organoids was significantly reduced compared with that in the central area of the organoids in both control JB6 T1-6 (Fig. 3d; tile_4, *P* = 0.0008; tile_5, *P* < 0.0001; tile_6, *P* < 0.0001; tile_7, *P* = 0.0012; tile_8-12, *P* = 0.0002) and *PRKN*-mutant PH13 T1-6 (Fig. 3d; tile_5, *P* = 0.0010; tile_6, *P* < 0.0001; tile_7,* P* < 0.0001; tile_8-12, *P* = 0.0011) *TH*-GFP iPSC lines. These results suggest that the neurites in the peripheral area of these organoids are mostly axons because axons extend longer than dendrites from the neuronal somata located in the organoid center. Immunostainings also demonstrated the presence of both TH-positive and TH-negative neurites in the peripheral area of the organoids (Fig. 3b, c). These results indicate that the peripheral area of *TH*-GFP iPSC-derived midbrain organoids is effective for the direct analysis of axonal mitochondria by live-cell imaging and subsequent electron microscopy, both in GFP-positive dopaminergic neurons and GFP-negative non-dopaminergic neurons.

### Membrane potential of axonal mitochondria in the peripheral area of *TH*-GFP iPSC-derived midbrain organoids

**Fig. 4:**
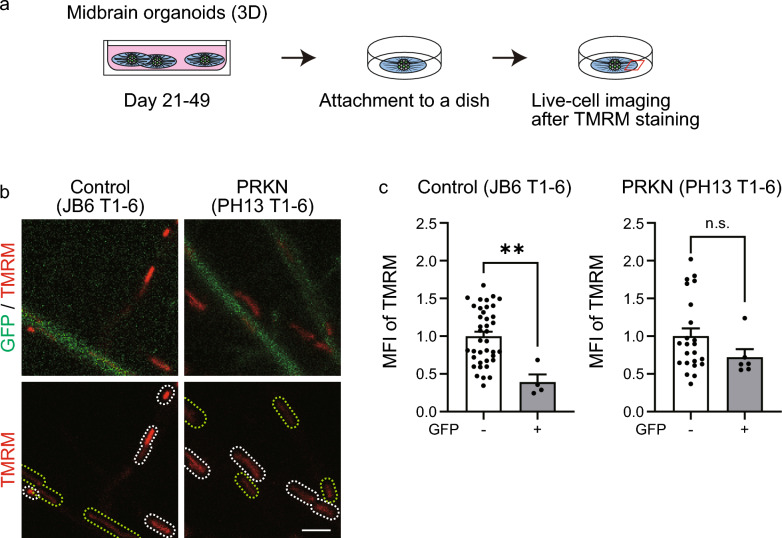
Analysis of mitochondrial membrane potential in axons of midbrain organoids differentiated from *TH*-GFP iPSCs. **a** Schematic timeline for live-cell imaging of midbrain organoids stained with TMRM. *TH*-GFP iPSC-derived midbrain organoids were cultured in ultralow-attachment plates for 4 weeks, followed by attachment to a dish and observation in the peripheral area of a midbrain organoid as indicated by the red square in the scheme. **b** Live-cell imaging of axons of GFP-positive dopaminergic neurons and GFP-negative non-dopaminergic neurons in midbrain organoids stained with TMRM. Green and white dotted lines indicate mitochondria in axons of dopaminergic and non-dopaminergic neurons, respectively. “PRKN” represents *PRKN*-mutant patient. Scale bar, 2 µm. **c** Mean TMRM MFI in all mitochondria for each axon from the live-cell images. Data were acquired from GFP-negative (n = 39) and GFP-positive (n = 4) axons from two fields of two organoids in the control JB6 T1-6 line and from GFP-negative (n = 23) and GFP-positive (n = 6) axons from two fields of two organoids in the *PRKN*-mutant PH13 T1-6 line. The graph shows the relative comparison of the mean TMRM MFI of mitochondria in GFP-negative and GFP-positive neurites. “PRKN” represents *PRKN*-mutant patient. Values are shown as the mean ± SEM. The differences were evaluated using the nonparametric Mann–Whitney U test. **P < 0.01. The mean TMRM MFI of mitochondria significantly differs between dopaminergic and non-dopaminergic neurons in the control JB6 T1-6 line (*P* = 0.002; effect size, 0.562; 95% CI, 0.384 to 0.872), whereas no significant difference is observable in the *PRKN*-mutant PH13 T1-6 line (*P* = 0.142; effect size, 0.177; 95% CI, − 0.0496 to 0.383). 3D, three-dimensional; CI, confidence interval; GFP, green fluorescence protein; iPSC, induced pluripotent stem cell; MFI, mean fluorescence intensity; n.s., not significant; SEM, standard error of the mean; TH, tyrosine hydroxylase; TMRM, tetramethylrhodamine methyl ester

To compare mitochondrial membrane potentials between axons of GFP-positive dopaminergic neurons and those of GFP-negative non-dopaminergic neurons, we attached organoids to dishes on day 49 and performed live-cell imaging of TMRM-stained organoids (Fig. 4a). Live-cell imaging was performed in the Tau-positive/MAP2-negative peripheral area, which primarily comprises axons (Fig. 3b, c, d). Because MitoTracker Deep Red insufficiently penetrated organoids, we identified mitochondria using the fluorescence intensity and size (in pixels) of TMRM itself to quantify mitochondrial membrane potentials in organoids. The quantitative analysis of these images showed that the TMRM intensity of mitochondria in GFP-positive neurites was significantly lower than that of mitochondria in GFP-negative neurites in both newly generated (Fig. 4b and c; JB6 T1-6, *P* = 0.002) and previously established (Fig. S3a and b in Additional file 1; control 201B7 T1-3, *P* = 0.0005; control WD39 T1-2, *P* = 0.0069) control *TH*-GFP iPSC lines. Although the axonal mitochondrial membrane potential was not significantly different between dopaminergic and non-dopaminergic neurons in patient lines (Fig. 4b and c; *PRKN*-mutant PH13 T1-6, *P* = 0.142; Fig. S3b in Additional file 1; *PRKN*-mutant PB2 T1-1, *P* = 0.665), they tended to show a similar difference as the control lines. These results indicate that axonal mitochondria in dopaminergic neurons have a lower membrane potential than those in non-dopaminergic neurons in the control *TH*-GFP iPSC lines. This finding is similar to the low mitochondrial membrane potential in the neurites (Fig. 2b and c, Fig. S2a and b in Additional file 1) and somata of 2D monolayer dopaminergic neurons [8].

### CLEM analysis of axonal mitochondria in the peripheral area of *TH*-GFP iPSC-derived midbrain organoids

**Fig. 5:**
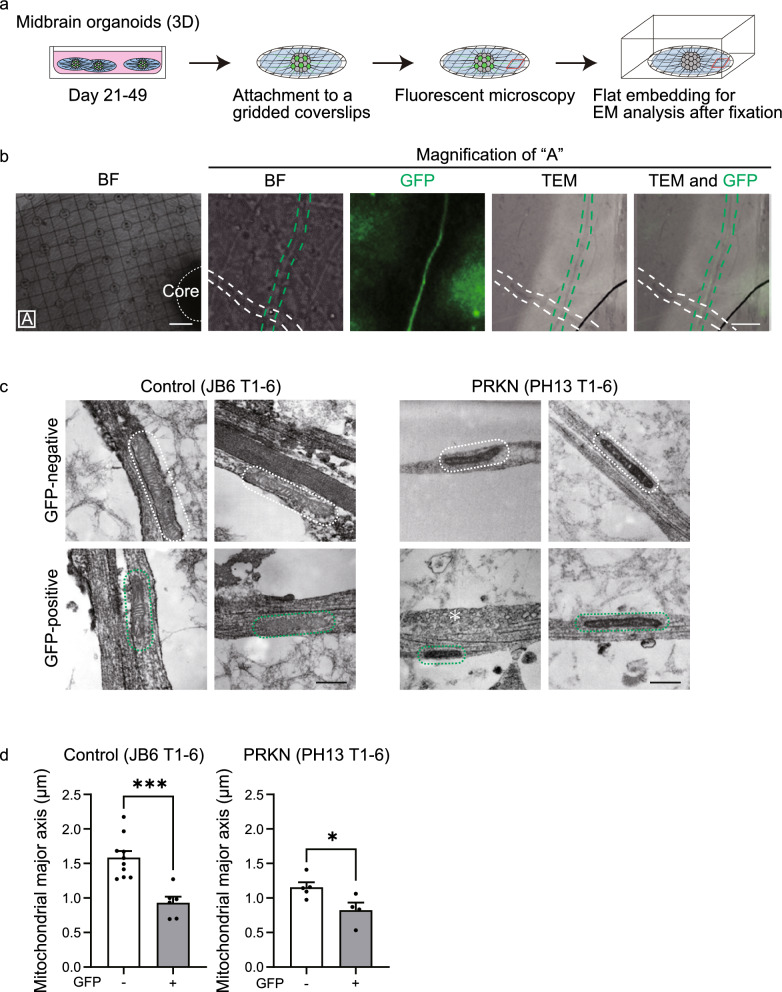
CLEM analysis of axonal mitochondria in midbrain organoids derived from *TH*-GFP iPSC lines. **a** Schematic timeline for CLEM analysis of axons in midbrain organoids derived from *TH*-GFP iPSC lines. Midbrain organoids were attached to gridded coverslips. After taking fluorescence images in the peripheral area (red square in the scheme) of the midbrain organoids, the midbrain organoids were fixed and flat-embedded for electron microscopic analysis. **b** Images of CLEM analysis of axons in midbrain organoids derived from *TH*-GFP iPSC lines. The leftmost panel shows a bright-field image of a midbrain organoid attached to a gridded coverslip. “A” indicates the peripheral area of the midbrain organoid. White dotted lines indicate the core of the midbrain organoid. Scale bar of the leftmost image, 100 µm. The remaining four panels are magnified images of “A,” showing from left to right bright-field, GFP, TEM, and merged TEM–GFP images. Green dotted lines indicate an axon of a GFP-positive dopaminergic neuron, and white dotted lines indicate an axon of a GFP-negative non-dopaminergic neuron. Scale bar of these four images, 5 µm. **c** TEM images of axonal mitochondria in midbrain organoids. White and green dotted lines indicate axonal mitochondria in GFP-negative non-dopaminergic and GFP-positive dopaminergic neurons, respectively. The asterisk indicates synaptic vesicles. “PRKN” represents *PRKN*-mutant patient. Scale bars, 500 nm. **d** Mean value of the major axis length in mitochondria per each axon from TEM images. Data were acquired from GFP-negative (n = 10) and GFP-positive (n = 6) axons in 33 TEM fields from one organoid derived from the control JB6 T1-6 line and from GFP-negative (n = 5) and GFP-positive (n = 4) axons from 39 TEM fields from one organoid derived from the *PRKN*-mutant PH13 T1-6 line. The lengths of mitochondrial major axes in TEM images were measured using ImageJ. “PRKN” represents *PRKN*-mutant patient. Values are shown as the mean ± SEM. The differences were evaluated using the nonparametric Mann–Whitney U test. *P < 0.05, ***P < 0.001. The mean of mitochondrial major axes significantly differs between dopaminergic and non-dopaminergic neurons in both the control JB6 T1-6 line (*P* = 0.0003; effect size, 0.605; 95% CI, 0.356 to 0.925) and the *PRKN*-mutant PH13 T1-6 line (*P* = 0.0317; effect size, 0.298; 95% CI, 0.0924 to 0.577). 3D, three-dimensional; BF, bright field; CLEM, correlative light–electron microscopy; CI, confidence interval; EM, electron microscopy; GFP, green fluorescence protein; iPSC, induced pluripotent stem cell; SEM, standard error of the mean; TEM, transmission electron microscope; TH, tyrosine hydroxylase

To investigate whether the morphology of axonal mitochondria differed between dopaminergic and non-dopaminergic neurons in correspondence with the observed differences in mitochondrial membrane potential, we attached organoids on gridded coverslips on day 49, captured bright-field and fluorescent images, and then fixed and embedded the organoids in resin for electron microscopy analyses (Fig. 5a). After identifying neurites in the peripheral area of the organoids in bright-field and fluorescent images on ultrathin sections (CLEM analysis, Fig. 5b), we observed mitochondria in the neurites using electron microscopy. After measuring the length of mitochondrial major axis in neurites, we used fluorescent images to confirm whether the neurites were GFP-positive or GFP-negative. CLEM analysis showed that mitochondria in GFP-positive neurites tended to be shorter in length than those in GFP-negative neurites in both control JB6 T1-6 and *PRKN*-mutant PH13 T1-6 *TH*-GFP iPSC lines (Fig. 5c). Quantitative analyses demonstrated that the mitochondrial major axis in GFP-positive neurites was significantly shorter in length than that in GFP-negative neurites in both the control JB6 T1-6 and *PRKN*-mutant PH13 T1-6 *TH*-GFP iPSC lines (Fig. 5d; control JB6 T1-6, *P* = 0.0003; *PRKN*-mutant PH13 T1-6, *P* = 0.032).

These results suggest that dopaminergic neurons have shorter mitochondria with lower membrane potential than non-dopaminergic neurons in the control iPSC line. Although the mitochondrial membrane potential did not significantly differ between dopaminergic and non-dopaminergic neurons in the *PRKN*-mutant patient iPSC line, *PRKN*-mutant patient dopaminergic neurons also tended to have shorter mitochondria with lower membrane potential compared to non-dopaminergic neurons, similar to the characteristics of somatic mitochondria in our previous study [8]. These findings indicate that *TH*-GFP iPSC-derived midbrain organoids can be a powerful tool to analyze mitochondrial function and morphology in the axons of human dopaminergic neurons.

## Discussion

In this study, we found that axons can be distinguished from dendrites in the peripheral area of midbrain organoids and that axonal mitochondria in dopaminergic neurons are characterized by shorter length and lower membrane potential than those in non-dopaminergic neurons.

Immunostainings demonstrated that the axonal marker Tau and the dendritic marker MAP2 colocalized in the neurites of 2D dopaminergic neurons differentiated for 28 days. In contrast, immunostainings of midbrain organoids differentiated for 49 days showed that most of the neurites in the peripheral area of the organoids were Tau-positive and MAP2-negative. Consistent with our findings, a previous study showed that terminal differentiation for more than two weeks restricted Tau and MAP2 signals to distinct cellular locations, suggesting that long-term culture induces neuronal polarity [27]. Furthermore, the extracellular 3D matrix influences lineage specification [28] and brain patterning [29]. In the present study, not only long-term culture but also the extracellular Geltrex surrounding the midbrain organoids may have promoted their neuronal polarity.

In this study, neurites in the peripheral area of the organoids mainly comprised axons of dopaminergic and non-dopaminergic neurons. Live-cell imaging and electron microscopy generally do not allow for immunostaining with antibodies against markers of neuronal polarity, making it difficult to identify axons among neurites. In the midbrain organoid model generated in this study, a neurosphere was embedded in the center of a Geltrex droplet, and neurites from the neurosphere extended radially along the Geltrex [16]. Therefore, we were able to identify axons in the peripheral area of the midbrain organoids (i.e., neurites in areas distant from the central aggregates of somata) without immunostaining for axonal markers. Furthermore, using *TH*-GFP iPSC lines to establish the midbrain organoid model enabled the analysis of the function and morphology of axonal mitochondria in GFP-positive dopaminergic neurons.

In the present study, live-cell imaging and CLEM analysis of the organoids demonstrated that axonal mitochondria in dopaminergic neurons were shorter with lower membrane potential than those in non-dopaminergic neurons. Similarly, somata of dopaminergic neurons in the substantia nigra of mice have smaller mitochondria than those of non-dopaminergic neurons [30]. We have also reported that somata of human iPSC-derived dopaminergic neurons have smaller mitochondria with lower membrane potential than those of non-dopaminergic neurons [8]. It remains unclear why dopaminergic neurons have smaller mitochondria with lower membrane potential. However, the axons of dopaminergic neurons in the substantia nigra are long and complex, and they have high energy demands [31, 32]. Dopaminergic neurons may shorten their mitochondria to facilitate the transport of mitochondria to these long and complex axons. The present study is the first to report that axons of human dopaminergic neurons have mitochondria with shorter length and lower membrane potential than axons of non-dopaminergic neurons.

Notably, the tendency for mitochondrial membrane potential to be lower in dopaminergic neurons was confirmed in all five *TH*-GFP iPSC lines. This characteristic of mitochondria in dopaminergic neurons, with their high energy demand but low membrane potential, might be one of the factors contributing to the predominant vulnerability of dopaminergic neurons in PD.

By contrast, in the *PRKN*-mutant patient lines, the differences in mitochondrial membrane potential and major axis length between dopaminergic and non-dopaminergic neurons were small or not significant. Likewise, the mitochondrial major axis length in somata did not significantly differ between dopaminergic and non-dopaminergic neurons in the *PRKN*-mutant patient iPSC line in our previous study [8]. One reason for the small difference between dopaminergic and non-dopaminergic neurons in *PRKN*-mutant patient iPSC lines might be that the *PRKN* mutation may have had a significant effect on the length and membrane potential of mitochondria in non-dopaminergic neurons. Our results indicate that short mitochondria with low membrane potential are a characteristic feature of dopaminergic neurons. However, this feature alone may not directly contribute to the pathogenesis of PD.

In this study, only mitochondrial membrane potential, one of the many mitochondrial functions, was measured using midbrain organoids. Recently, we have reported that the amount of contact sites between the endoplasmic reticulum and mitochondria is reduced in somata of *PRKN*-mutant patient dopaminergic neurons [33]. Endoplasmic reticulum–mitochondrial contact sites within axons are involved in axon regeneration in mice [34] and axon integrity in *Drosophila* [35]. In addition, intracellular ATP levels are reduced in *PRKN*-mutant patient iPSC-derived glutamatergic neurons [36]. Therefore, changes in endoplasmic reticulum–mitochondrial contact sites or ATP levels in axons of *PRKN*-mutant patient dopaminergic neurons may contribute to the development of PD. Using *TH*-GFP iPSC-derived midbrain organoids, it is also possible to analyze endoplasmic reticulum–mitochondrial contact sites or ATP levels in axons of *PRKN*-mutant patient dopaminergic neurons. Further analyses of endoplasmic reticulum–mitochondrial contact sites and ATP levels in the axons of dopaminergic neurons may help clarify the mechanisms of dopaminergic neuron degeneration in patients carrying *PRKN* mutations.

A limitation of this study is that the axons in the peripheral area of the midbrain organoids did not connect to other neurons. Recently, midbrain–striatum assembloids have been reported, which fuse midbrain organoids with striatal organoids [37, 38]. Instead of fusing organoids, a new method called connectoid has also been reported, in which two organoids are placed in a microfluidic channel, and the organoids, separated from each other, connect by extending their axons [39]. The axons in the peripheral area of the midbrain organoids used in the present study do not have an integrated synaptic network. Thus, the generation of assembloids or connectoids might enable the analysis of axonal mitochondria within an integrated neural network. Furthermore, connectoids between midbrain and striatal organoids would clarify the properties of axonal mitochondria in functional nigrostriatal networks.

In conclusion, this study demonstrated that the peripheral area of midbrain organoids allowed identifying the characteristics of axonal mitochondria in dopaminergic neurons. The findings about axonal mitochondria in dopaminergic neurons will help elucidate the mechanisms of dopaminergic neuron degeneration in patients with *PRKN* mutations.

## Supplementary Information

Additional file1. PCR analysis of knock-in iPSC clones with TH-GFP alleles. The TH-GFP allele produced by insertion of the GFP gene into the targeted site is detected as a 1.5 kb band. “T1” represents knock-in iPSCs at the target site. The numbers 3, 5, 6, 7 and 3, 6 indicate the knock-in clone numbers derived from the control JB6 and PRKN-mutant PH13 iPSC lines, respectively. GFP: green fluorescence protein, iPSC: induced pluripotent stem cell, PCR: polymerase chain reaction, TH: tyrosine hydroxylase

Additional file2. Analysis of mitochondrial membrane potential in neurites derived from other TH-GFP iPSC lines. (a) Live-cell imaging of neurites of dopaminergic and non-dopaminergic neurons derived from other TH-GFP iPSC lines. Green and white dotted lines indicate mitochondria in GFP-positive and GFP-negative neurites, respectively. “PRKN” represents PRKN-mutant patient. Scale bar, 2 µm. (b) Mean TMRM MFI in all mitochondria per neurite from the live-cell images. Data were acquired from GFP-negative (n = 20) and GFP-positive (n = 12) neurites from two fields in the control 201B7 T1-3 line, from GFP-negative (n = 19) and GFP-positive (n = 6) neurites from two fields in the control WD39 T1-2 line, and from GFP-negative (n = 16) and GFP-positive (n = 8) neurites from two fields in the PRKN-mutant PB2 T1-1 line. The graph shows the relative comparison of the mean TMRM MFI of mitochondria in GFP-negative and GFP-positive neurites. “PRKN” represents PRKN-mutant patient. Values are shown as the mean ± SEM. The differences were evaluated using the nonparametric Mann–Whitney U test. **P < 0.01, ***P < 0.001. The mean TMRM MFI of mitochondria significantly differs between dopaminergic and non-dopaminergic neurons in the control lines B7 T1-3 (P = 0.0126; effect size, 0.383; 95% CI, 0.185 to 0.592) and WD39 T1-2 (P = 0.0002; effect size, 0.585; 95% CI, 0.336 to 0.816), whereas no significant difference is observable in the PRKN-mutant PB2 T1-1 line (P = 0.569; effect size, 0.0783; 95% CI, −0.156 to 0.419). BF: bright field, CI: confidence interval, GFP: green fluorescence protein, iPSC: induced pluripotent stem cell, MFI: mean fluorescence intensity, n.s.: not significant, SEM: standard error of the mean, TH: tyrosine hydroxylase, TMRM: tetramethylrhodamine methyl ester

Additional file3. Analysis of mitochondrial membrane potential in axons of midbrain organoids derived from other TH-GFP iPSC lines. (a) Live-cell imaging of axons of GFP-positive dopaminergic and GFP-negative non-dopaminergic neurons in midbrain organoids stained with TMRM. Green and white dotted lines indicate mitochondria in axons of dopaminergic and non-dopaminergic neurons, respectively. “PRKN” represents PRKN-mutant patient. Scale bar, 2 µm. (b) Mean TMRM MFI in all mitochondria per each axon from live-cell images. Data were acquired from GFP-negative (n = 42) and GFP-positive (n = 8) axons from two fields of two organoids in the control B7 T1-3 line, from GFP-negative (n = 21) and GFP-positive (n = 3) axons from two fields of two organoids in the control WD39 T1-2 line, and from GFP-negative (n = 68) and GFP-positive (n = 3) axons from two fields of two organoids in the PRKN-mutant PB2 T1-1 line. The graph shows the relative comparison of the mean TMRM MFI of mitochondria in GFP-negative and GFP-positive neurites. “PRKN” represents PRKN-mutant patient. Values are shown as the mean ± SEM. The differences were evaluated using the nonparametric Mann–Whitney U test. **P < 0.01, ***P < 0.001. The mean TMRM MFI of mitochondria significantly differs between dopaminergic and non-dopaminergic neurons in the control line 201B7 T1-3 (P = 0.0005; effect size, 0.329; 95% CI, 0.207 to 0.622) and WD39 T1-2 (P = 0.0069; effect size, 0.578; 95% CI, 0.372 to 0.772), whereas no significant difference is observable in the PRKN-mutant PB2 T1-1 line (P = 0.486; effect size, 0.124, 95% CI, −0.225 to 0.329). CI: confidence interval, GFP: green fluorescence protein, iPSC: induced pluripotent stem cell, MFI: mean fluorescence intensity, n.s.: not significant, SEM: standard error of the mean, TH: tyrosine hydroxylase, TMRM: tetramethylrhodamine methyl ester

## Data Availability

The datasets used and/or analyzed during the current study are available from the corresponding author upon reasonable request.

## Abbreviations

2D: Two-dimensional
3D: Three-dimensional
BSA: Bovine serum albumin
CLEM: Correlative light–electron microscopy
DAPI: 4′,6-Diamidino-2-phenylindole
GFP: Green fluorescence protein
iPSC: Induced pluripotent stem cell
MAP2: Microtubule-associated protein 2
MFI: Mean fluorescence intensity
PBS: Phosphate-buffered saline
PD: Parkinson’s disease
PRKN: Parkin
sgRNA: Single-guide RNA
TH: Tyrosine hydroxylase
TMRM: Tetramethylrhodamine methyl ester
