# Supplementary figures and images for: Human midbrain organoids reveal the characteristics of axonal mitochondria specific to dopaminergic neurons
Source: Mol Brain. 2025 Dec 25;19:8. doi: 10.1186/s13041-025-01268-w (PMC12849627; doi:10.1186/s13041-025-01268-w)

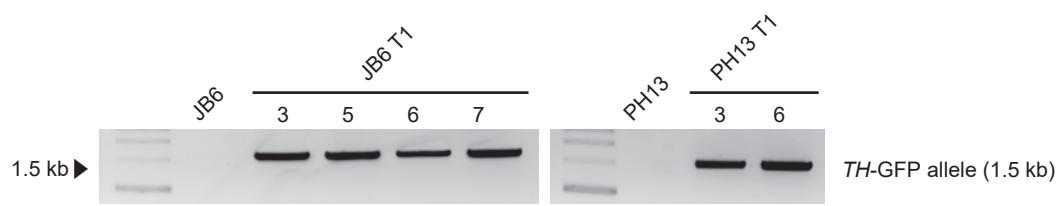

Supplement: Supplementary file 1 — Additional file1. PCR analysis of knock-in iPSC clones with TH-GFP alleles. The TH-GFP allele produced by insertion of the GFP gene into the targeted site is detected as a 1.5 kb band. “T1” represents knock-in iPSCs at the target site. The numbers 3, 5, 6, 7 and 3, 6 indicate the knock-in clone numbers derived from the control JB6 and PRKN-mutant PH13 iPSC lines, respectively. GFP: green fluorescence protein, iPSC: induced pluripotent stem cell, PCR: polymerase chain reaction, TH: tyrosine hydroxylase [file 13041_2025_1268_MOESM1_ESM.pdf]

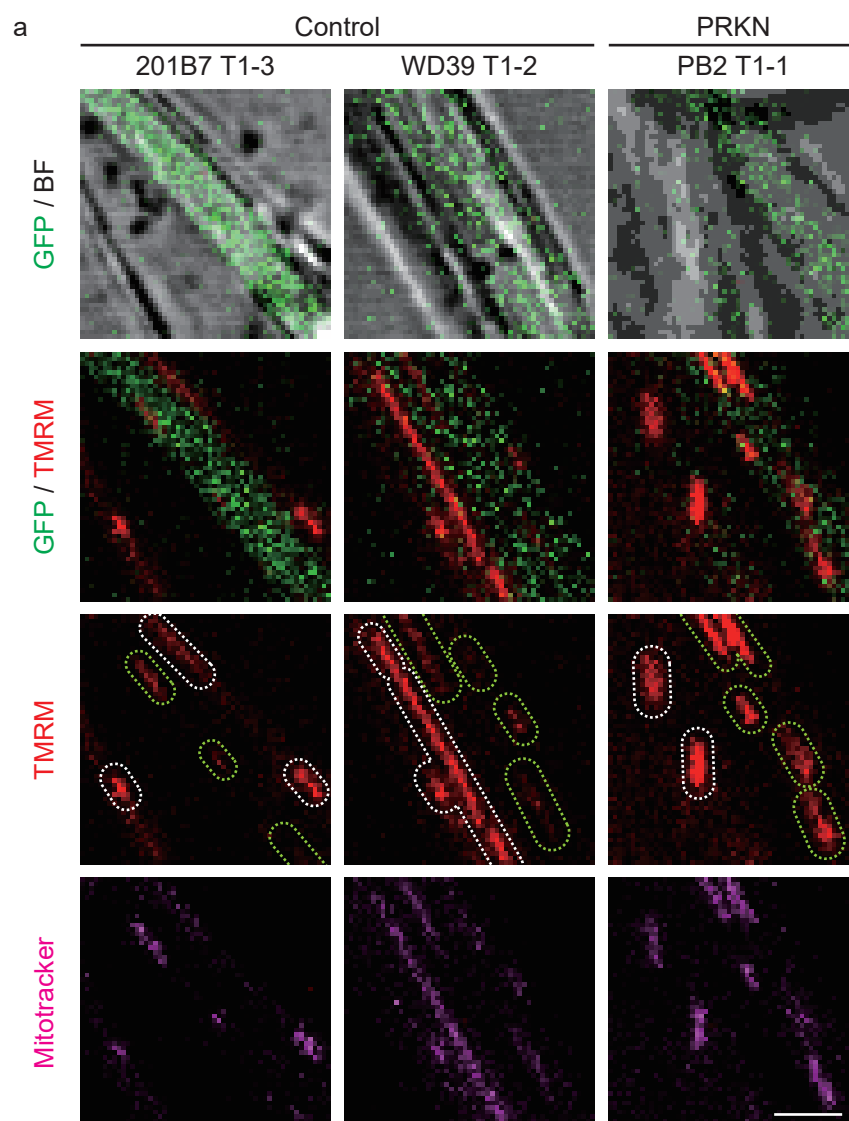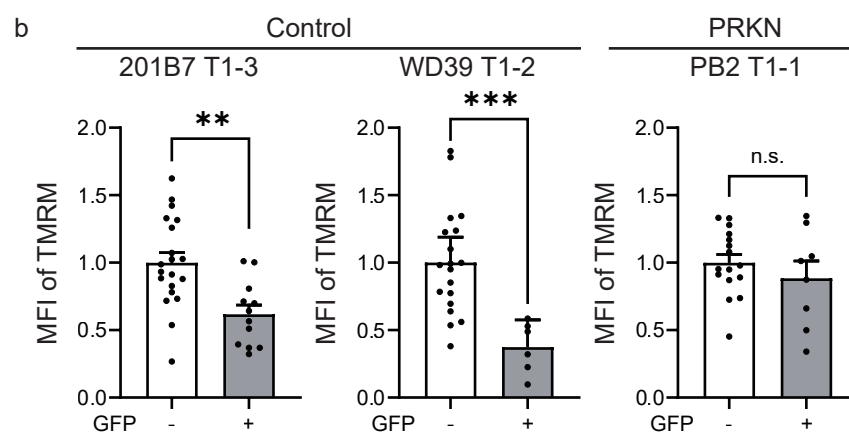

Supplement: Supplementary file 2 — Additional file2. Analysis of mitochondrial membrane potential in neurites derived from other TH-GFP iPSC lines. (a) Live-cell imaging of neurites of dopaminergic and non-dopaminergic neurons derived from other TH-GFP iPSC lines. Green and white dotted lines indicate mitochondria in GFP-positive and GFP-negative neurites, respectively. “PRKN” represents PRKN-mutant patient. Scale bar, 2 µm. (b) Mean TMRM MFI in all mitochondria per neurite from the live-cell images. Data were acquired from GFP-negative (n = 20) and GFP-positive (n = 12) neurites from two fields in the control 201B7 T1-3 line, from GFP-negative (n = 19) and GFP-positive (n = 6) neurites from two fields in the control WD39 T1-2 line, and from GFP-negative (n = 16) and GFP-positive (n = 8) neurites from two fields in the PRKN-mutant PB2 T1-1 line. The graph shows the relative comparison of the mean TMRM MFI of mitochondria in GFP-negative and GFP-positive neurites. “PRKN” represents PRKN-mutant patient. Values are shown as the mean ± SEM. The differences were evaluated using the nonparametric Mann–Whitney U test. **P < 0.01, ***P < 0.001. The mean TMRM MFI of mitochondria significantly differs between dopaminergic and non-dopaminergic neurons in the control lines B7 T1-3 (P = 0.0126; effect size, 0.383; 95% CI, 0.185 to 0.592) and WD39 T1-2 (P = 0.0002; effect size, 0.585; 95% CI, 0.336 to 0.816), whereas no significant difference is observable in the PRKN-mutant PB2 T1-1 line (P = 0.569; effect size, 0.0783; 95% CI, −0.156 to 0.419). BF: bright field, CI: confidence interval, GFP: green fluorescence protein, iPSC: induced pluripotent stem cell, MFI: mean fluorescence intensity, n.s.: not significant, SEM: standard error of the mean, TH: tyrosine hydroxylase, TMRM: tetramethylrhodamine methyl ester [file 13041_2025_1268_MOESM2_ESM.pdf]

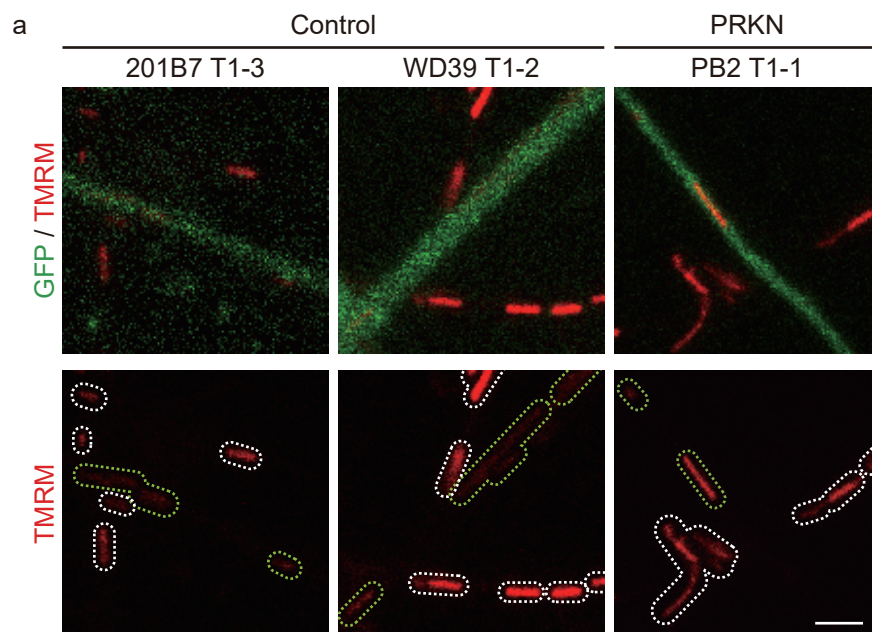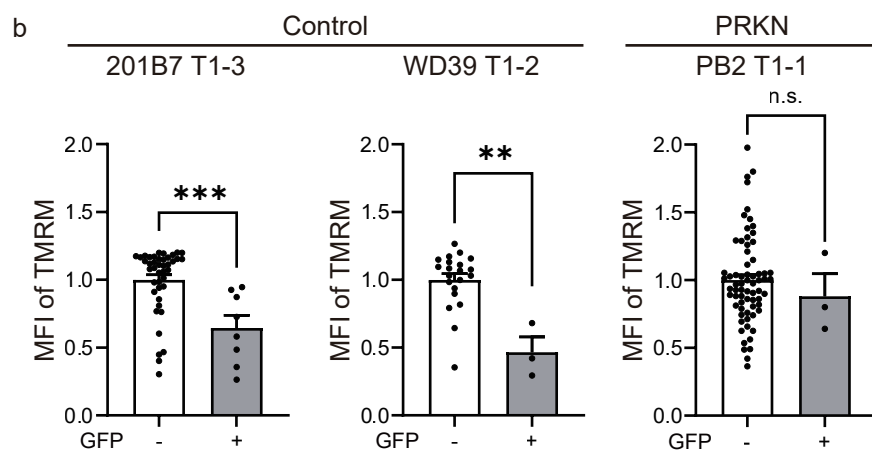

Supplement: Supplementary file 3 — Additional file3. Analysis of mitochondrial membrane potential in axons of midbrain organoids derived from other TH-GFP iPSC lines. (a) Live-cell imaging of axons of GFP-positive dopaminergic and GFP-negative non-dopaminergic neurons in midbrain organoids stained with TMRM. Green and white dotted lines indicate mitochondria in axons of dopaminergic and non-dopaminergic neurons, respectively. “PRKN” represents PRKN-mutant patient. Scale bar, 2 µm. (b) Mean TMRM MFI in all mitochondria per each axon from live-cell images. Data were acquired from GFP-negative (n = 42) and GFP-positive (n = 8) axons from two fields of two organoids in the control B7 T1-3 line, from GFP-negative (n = 21) and GFP-positive (n = 3) axons from two fields of two organoids in the control WD39 T1-2 line, and from GFP-negative (n = 68) and GFP-positive (n = 3) axons from two fields of two organoids in the PRKN-mutant PB2 T1-1 line. The graph shows the relative comparison of the mean TMRM MFI of mitochondria in GFP-negative and GFP-positive neurites. “PRKN” represents PRKN-mutant patient. Values are shown as the mean ± SEM. The differences were evaluated using the nonparametric Mann–Whitney U test. **P < 0.01, ***P < 0.001. The mean TMRM MFI of mitochondria significantly differs between dopaminergic and non-dopaminergic neurons in the control line 201B7 T1-3 (P = 0.0005; effect size, 0.329; 95% CI, 0.207 to 0.622) and WD39 T1-2 (P = 0.0069; effect size, 0.578; 95% CI, 0.372 to 0.772), whereas no significant difference is observable in the PRKN-mutant PB2 T1-1 line (P = 0.486; effect size, 0.124, 95% CI, −0.225 to 0.329). CI: confidence interval, GFP: green fluorescence protein, iPSC: induced pluripotent stem cell, MFI: mean fluorescence intensity, n.s.: not significant, SEM: standard error of the mean, TH: tyrosine hydroxylase, TMRM: tetramethylrhodamine methyl ester [file 13041_2025_1268_MOESM3_ESM.pdf]
